# TRPC5 channels participate in pressure-sensing in aortic baroreceptors

**DOI:** 10.1038/ncomms11947

**Published:** 2016-07-14

**Authors:** On-Chai Lau, Bing Shen, Ching-On Wong, Yung-Wui Tjong, Chun-Yin Lo, Hui-Chuan Wang, Yu Huang, Wing-Ho Yung, Yang-Chao Chen, Man-Lung Fung, John Anthony Rudd, Xiaoqiang Yao

**Affiliations:** 1 Li Ka Shing Institute of Health Sciences, Faculty of Medicine, The Chinese University of Hong Kong, Hong Kong, China; 2 School of Biomedical Sciences, Faculty of Medicine, The Chinese University of Hong Kong, Hong Kong, China; 3 Department of Physiology, Anhui Medical University, Hefei 230032, China; 4 Department of Physiology, University of Hong Kong, Hong Kong, China

## Abstract

Blood pressure is maintained within a normal physiological range by a sophisticated regulatory mechanism. Baroreceptors serve as a frontline sensor to detect the change in blood pressure. Nerve signals are then sent to the cardiovascular control centre in the brain in order to stimulate baroreflex responses. Here, we identify TRPC5 channels as a mechanical sensor in aortic baroreceptors. In *Trpc5* knockout mice, the pressure-induced action potential firings in the afferent nerve and the baroreflex-mediated heart rate reduction are attenuated. Telemetric measurements of blood pressure demonstrate that *Trpc5* knockout mice display severe daily blood pressure fluctuation. Our results suggest that TRPC5 channels represent a key pressure transducer in the baroreceptors and play an important role in maintaining blood pressure stability. Because baroreceptor dysfunction contributes to a variety of cardiovascular diseases including hypertension, heart failure and myocardial infarction, our findings may have important future clinical implications.

## Abstract (web summary)

The identity of mechanosensors within aortic baroreceptors that sense fluctuations in blood pressure is unclear. Here, Lau *et al.* show that a cation channel TRPC5 acts as a transducer of mechanical stimuli in aortic baroreceptor neurons in rodents.

## Graphical abstract

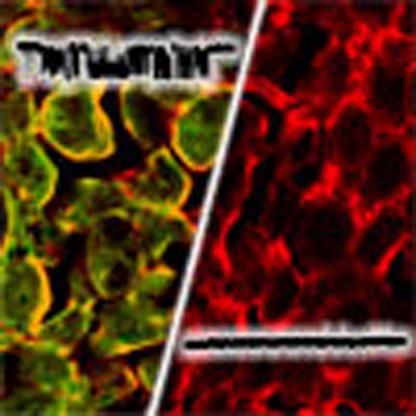

Blood pressure in the human body needs to be maintained within a relatively narrow physiological range. Fluctuation of blood pressure outside the normal physiological range may result in posture hypotension, strokes, heart attacks and aneurysms^1^,^2^,^3^. Changes in blood pressure are sensed by the baroreceptors at the sensory nerve terminals innervating aortic arch and carotid sinus. The mechanical signals are then converted into an action potential frequency, which propagates along the afferent fibres (aortic depressor nerve and carotid sinus nerve) to the somata located in the nodose and petrosal ganglions and subsequently to the cardiovascular control centre in the brainstem, causing reflex adjustments in heart rate, heart contractility and vascular tone, which eventually restore the blood pressure to normal levels^1^. Dysfunction of baroreceptors contributes to several cardiovascular diseases including hypertension and myocardial infarction^2^,^3^. Several studies have explored the molecular nature of baroreceptor mechanosensors and suggested the participation of several ion channels/components, including acid-sensing ion channel 2 (ASIC2), epithelial sodium channel γ-subunit and TRPV1 in baroreceptor functions^4^,^5^,^6^. However, these findings are controversial^7^,^8^. Until now, the molecular identity of the aortic arch baroreceptor mechanosensors remains elusive.

Transient receptor potential (TRP) channels are a group of cation channels that act as cellular sensors to perceive and respond to a variety of environmental stimuli including temperature, taste and pain^9^. Multiple TRP channels have also been shown to be sensitive to various forms of mechanical stimuli including direct membrane stretching, hypoosmolarity and fluid shear stress^10^. Membrane stretching activates TRPC5, -V2, -V4, -M4 and -M7 directly^10^,^11^,^12^,^13^,^14^,^15^, whereas hydrostatic pressure activates TRPC6 and -M4 indirectly through cytosolic signalling molecules^10^. Among these channels, the membrane stretch-activated channels are especially important, because they can directly sense mechanical stimuli and then transform the mechanical signals into electrical currents. Such properties make these channels attractive candidates as mechanosensors and mechanotransducers. Previous studies have demonstrated that TRPC5 is activated by hydrostatic pressure, hypoosmolarity and membrane stretching^13^,^14^,^15^. However, the physiological function of TRPC5 as a mechanosensor is unknown. Notably, TRPC5 is reported to be expressed in baroreceptor neurons^16^,^17^.

In the present study, we hypothesize that TRPC5 acts as a pressure sensor in baroreceptor neurons. A stretch-activated channel was recorded in the aortic baroreceptor neurons whose properties resemble TRPC5. In *Trpc5*^*−/−*^ mice, the pressure-elicited activation of aortic depressor nerve and carotid sinus nerve activities was reduced, and the baroreflex-mediated heart rate response was attenuated. *Trpc5*^*−/−*^ mice also displayed instability in daily blood pressure. These data suggest an important role of TRPC5 in baroreceptor mechanosensation.

## Results

### Stretch activation of TRPC5 at the single-channel level

**Figure 1:**
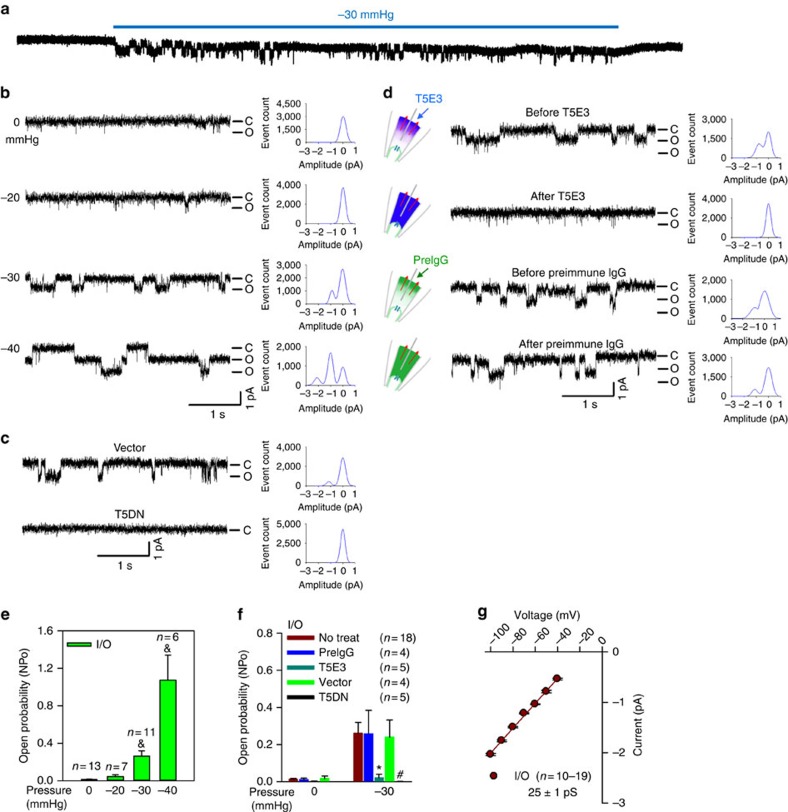
A stretch-activated TRPC5-like channel in rat baroreceptor neurons. Representative traces (**a-d**), their corresponding event histograms (**b-d**) and summarized single-channel open probabilities (NP_o_,** e-f**) showing a stretch-activated channel. The recordings were made in excised inside-out patches (i/o) from the neurite terminals of DiI-labelled primary cultured aortic baroreceptor neurons with a holding potential at −60 mV. (**a**) Continuous recording of single-channel current trace before and after negative pressure of −30 mm Hg. (**b**,**e**) Representative traces from a single patch under different pressures (**b**) and data summary (**e**). (**c**,**d**,**f**) Effect of T5DN (**c** and **f**, vector as control) and T5E3 (**d** and **f**, preimmune IgG as control) on the channel activities at −30 mm Hg pressure. NP_o_ were summarized from multiple patches. (**g**) Single-channel *I*−*V* relationship of the stretch-activated channel. Mean±s.e.m. ^&^*P*<0.05 as compared with 0 mm Hg, *P<0.05 as compared with preimmune IgG, ^#^*P*<0.05 as compared with vector, statistical analysis was performed by Student’s *t*-test.

Patch clamp recording was used to study the stretch-activated channels in the neurite terminals of primary cultured aortic baroreceptor neurons (Fig. 1). The aortic baroreceptor neurons were isolated from the left nodose ganglion and identified by 1,1'-dioleyl-3,3,3′,3′-tetramethylindocarbocyanine methanesulfonate (DiI) labelling^18^,^19^. The DiI-positive baroreceptor neurons were selected for functional studies. Figure 1a illustrates the patterns of single-channel activities recorded in a typical excised inside-out patch from the neurite terminals at −60 mV holding potential. The pipette solution contained mainly normal physiological saline solution, and the bath solution was mainly Cs-aspartate. 20 μmol l^−1^ La^3+^ was included in the pipette solution to potentiate TRPC5 activity and block other cation channels^20^. No channel activity was observed in the absence of pipette pressure. When the negative suction in the pipette reached −30 mm Hg and −40 mm Hg, the channel activity increased drastically (Fig. 1a,b,e). Approximately 80% of patches (118 out of 148 patches) contained mechanosensitive channels. This stretch-activated channel could also be recorded in the cell-attached mode from the neurite terminals and the somata of baroreceptor neurons (Supplementary Fig. 1). Single-channel slope conductance was estimated to be 25±1 pS (*n*=19) from inside-out patches of neurite terminals (Fig. 1g) and 26±2 pS from cell-attached patches of somata (Supplementary Fig. 1f).

To confirm the involvement of TRPC5, we backfilled the patch pipettes with a TRPC5 blocking antibody (T5E3)^21^ using a two-step protocol^22^. In this design, T5E3 would slowly diffuse from the pipette onto the membrane, causing a time-dependent inhibition of TRPC5. In both inside-out and cell-attached patches from neurite terminals, T5E3 (15 μg ml^−1^) inhibited the stretch-activated single-channel activity, whereas preimmune IgG (PreIgG) had no effect (Fig. 1d,f and Supplementary Fig. 1c). T5E3 also had an inhibitory effect on the cell-attached patches on the somata (Supplementary Fig. 1e). A dominant-negative TRPC5 construct (T5DN) was used to disrupt the function of endogenous TRPC5 in baroreceptor neurons^23^. In T5DN-transfected neurons, the stretch-activated channel was absent (Fig. 1c,f and Supplementary Fig. 1c,e). These data strongly suggest that the stretch-activated channel in the aortic baroreceptor neurons contains TRPC5.

### Role of TRPC5 in hypoosmolarity-activated whole-cell current

**Figure 2:**
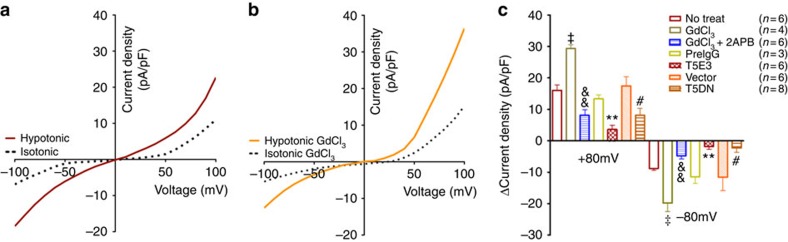
TRPC5 channel in the hypertonicity-induced whole-cell current. (**a**,**b**) Whole-cell *I–V* relationships of a representative single primary cultured aortic baroreceptor neurons in isotonic (300 mOsm) and hypotonic (240 mOsm) conditions in the absence (**a**) and presence (**b**) of 20 μmol l^−1^ GdCl_3_. *I*–*V* relationship was obtained using a ramp protocol from −100 mV to +100 mV with 500-ms duration and repeated every 1 s. (**c**) Summary of data showing the hypoosmolarity-activated whole-cell current density at ±80 mV under different conditions. Subtraction of currents in isotonic condition from the hypotonic one yielded the hypoosmolarity-activated currents (Δ current density). T5E3, 15 μg ml^−1^; T5DN, T5DN transfection; 2-APB, 100 μmol l^−1^. Mean±s.e.m. ^‡^*P*<0.01 as compared with no-treatment, ^&&^*P*<0.01 as compared with GdCl_3_, ^**^P<0.01 as compared with preimmune IgG and ^*#*^*P*<0.05 as compared with vector, statistical analysis was performed by Student’s *t*-test.

The role of TRPC5 in baroreceptor mechanosensing was also studied at whole-cell level. In the cultured aortic baroreceptor neurons, hypoosmolarity activated a whole-cell cationic current (Fig. 2a,b), which was potentiated by Gd^3+^ (20 μmol l^−1^, Fig. 2b,c), but was suppressed by T5E3, T5DN and a TRPC inhibitor 2-aminoethoxydiphenyl borate (2-APB; Fig. 2c). The current–voltage relationships (*I*−*V*) of hypoosmolarity-activated current, especially those in the presence of Gd^3+^, displayed double rectification typical of TRPC5 (Fig. 2b)^20^.

We also explored the possible role of other TRPC isoforms in baroreceptor mechanosensing at whole-cell level. Short interfering RNAs (siRNAs) were used to knock-down *Trpc1*, *Trpc3*, *Trpc4*, *Trpc5* and *Trpc6* mRNA (Supplementary Fig. 2). Among them, only knockdown of *Trpc5* mRNA resulted in a significant reduction in pressure-activated whole-cell current in rat aortic baroreceptor neurons (Supplementary Fig. 2c).

### Endogenous TRPC5 expression in rat aortic baroreceptor neurons

**Figure 3:**
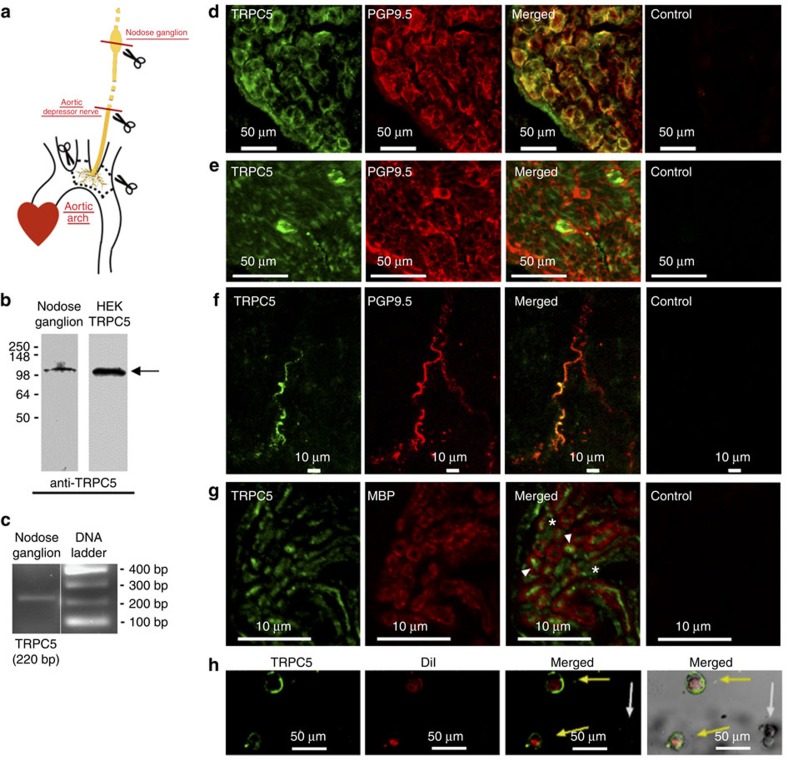
Endogenous TRPC5 expression in rat aortic baroreceptor neurons. (**a**) A schematic diagram showing the positions of left nodose ganglion, aortic depressor nerve and baroreceptor terminals in aortic arch. Tissue sections and aortic arch adventitia were obtained at the positions as indicated by *dashes* and *scissors*. (**b**) Representative images of immunoblots (*n*=3) showing endogenous expression of TRPC5 proteins in left nodose ganglion (left lane). Lysate from TRPC5-overexpressing HEK293 cells were used as positive control (right lane). (**c**) RT–PCR detection of *Trpc5* mRNA in left nodose ganglion. Shown were the RT–PCR products of *Trpc5* and the DNA ladder (*n*=3). (**d–f**) Representative images (*n*=3) showing the immunoreactivity to TRPC5 (green), a neuronal marker PGP9.5 (red, **d–f**) at the left nodose ganglion (**d**), aortic depressor nerve (**e**) and baroreceptor terminals within the aortic arch adventitia (**f**). Also shown were the merged images of TRPC5 and PGP9.5. (**g**) Representative images (*n*=3) showing the immunoreactivity to TRPC5 (green) and a myelin-marker myelin basic protein (MBP) at aortic depressor nerve. Controls were without primary antibodies. In the merged image, *arrowheads* show the TRPC5 expression in myelinated fibre, whereas *asterisks* illustrate the TRPC5 expression in unmyelinated fibres. (**h**) T5E3 labelling (green) of DiI-positive (red) aortic baroreceptor neurons (*n*=4). These were non-permeabilized neurons in primary culture.

The immunoreactive signals of TRPC5 proteins were detected in the somata of sensory neurons in rat left nodose ganglion, the peripheral axons of these neurons in the aortic depressor nerve and the baroreceptor terminals that innervate the aortic arch adventitia (Fig. 3a,d–g). The signals of TRPC5 proteins localized closely with that of the neuronal marker PGP9.5 (Fig. 3d-f). 70–80% neurons were TRPC5-positive. TRPC5 expression was found in myelinated and some unmyelinated axon fibres (Fig. 3g). The expressions of TRPC5 proteins and mRNAs in rat nodose ganglion neurons were confirmed by immunoblots and reverse transcriptase PCR (RT–PCR), respectively (Fig. 3b,c). TRPC5 proteins were present in the plasma membrane, because T5E3, which is an extracellular epitope-targeting antibody, was able to stain the non-permeabilized baroreceptor neurons (Fig. 3h).

### Role of TRPC5 in baroreceptor mechanosensation in rats

**Figure 4:**
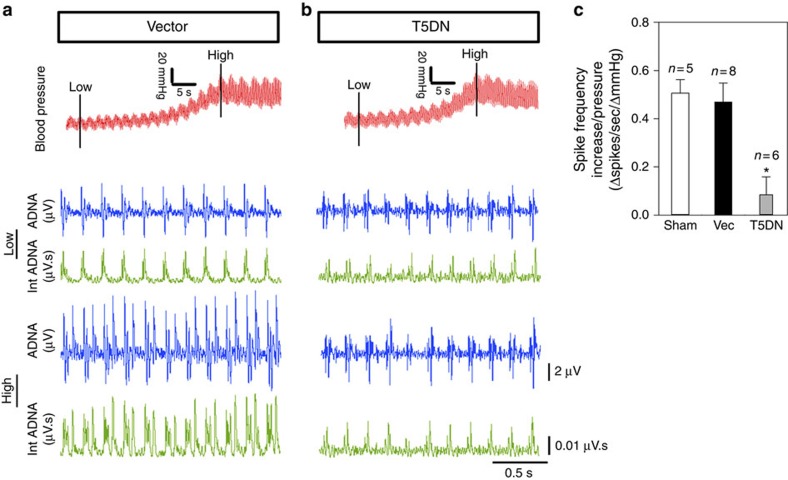
TRPC5 participates in the pressure-sensing of rat aortic baroreceptors. (**a**,**b**) Representative traces showing that blood pressure elevation (upper row) increased the spike frequency and magnitude of aortic depressor nerve activity (ADNA) in the left aortic depressor nerve. Integrated ADNA (Int ADNA) are also shown. The middle two rows show the spike traces and Int ADNA at low blood pressure, whereas the bottom two rows show the spike traces and Int ADNA at high blood pressure. Rats were infected with either lenti-vector (**a**) or lenti-T5DN (**b**) at the nodose ganglion. (**c**) Data summary comparing the baroreceptor sensitivity (Δspikes s^−1^ ΔmmHg^−1^), which is the change in spike frequency per mmHg pressure increase. Sham, sham-operated; Vec, vector. Mean±s.e.m. (*n*=6–8). *P<0.05 as compared with vector, statistical analysis was performed by Student’s *t*-test.

To test the involvement of TRPC5 in rat baroreceptor mechanosensation *in vivo*, we monitored the frequency of the action potential discharge (spike frequency) in the left aortic depressor nerve as well as the baroreflex regulation of heart rate. We developed a lentivirus-mediated *in vivo* transgene delivery to nodose ganglion in order to disrupt the function of endogenous TRPC5. The left nodose ganglion of the rats was infected with T5DN-carrying lentivirus (lenti-T5DN) or empty lentiviral vector (lenti-vector) as control. The transduction efficiency was verified by transducing nodose ganglion with the lentivirus carrying green fluorescent protein (GFP). GFP fluorescence was observed in 82% of ganglion neurons and also at the nerve terminals in aortic arch (Supplementary Fig. 3). Elevation of arterial blood pressure by an inflatable cuff around the abdominal aorta increased the spike frequency and the magnitude of integrated aortic depressor nerve activity (Int ADNA) in control rats that were infected with lenti-vector (Fig. 4a). In contrast, the pressure-induced increases in spike frequency and Int ADNA were much smaller in rats infected with lenti-T5DN than those infected with lenti-vector or sham-operated rats (Fig. 4b,c). Baroreceptor sensitivity (Δspikes s^−1^ ΔmmHg^−1^) was also clearly reduced in rats infected with lenti-T5DN (Fig. 4c).

Baroreflex control of heart rate was also examined in S/D rats. Acute intravenous infusion of phenylephrine into femoral vein at the range of 2.5–30 μg min^−1^ produced a dose-dependent and transient increase in the mean arterial pressure and a consequent fall in heart rate, reflecting the baroreflex control of heart rate (Supplementary Fig. 4). In rats infected with lenti-T5DN, the phenylephrine-induced heart rate reduction was markedly attenuated (Supplementary Fig. 4).

### Deficiency of baroreceptor function in *Trpc5* knockout (*Trpc5* ^*−/−*^) mice

**Figure 5:**
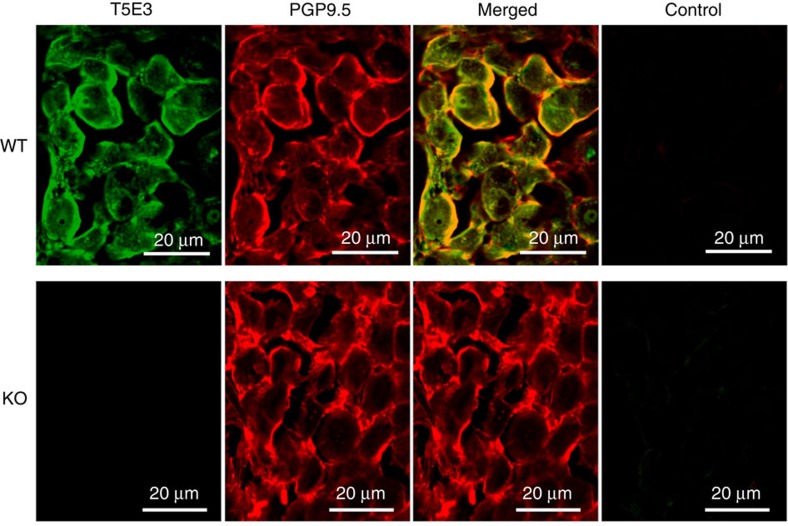
Endogenous TRPC5 expression in mouse aortic baroreceptor neurons. Immunoreactivities to TRPC5 using T5E3 as the primary antibody (green), and to a neuronal marker PGP9.5 (red), on non-permeabilized tissue slides from nodose ganglion of wild-type (WT) and *Trpc5*^*−/−*^ (KO) mice. Also shown are the merged images and control images. The control contained no primary antibodies. *n*=3 experiments.

**Figure 6:**
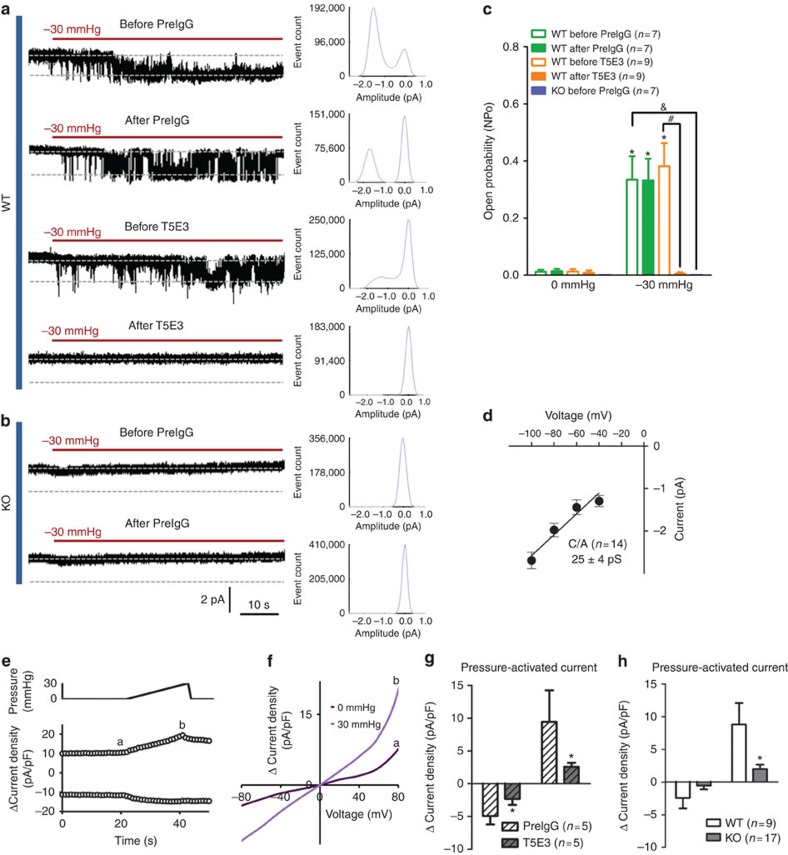
Comparison of TRPC5-like channel between wild-type and *Trpc5*^*−/−*^ mice. (**a–c**) Representative traces (**a**,**b**), their corresponding event histogram (**a**,**b**) and summarized data (**c**) showing a stretch-activated channel in cell-attached patches (c/a) from the somata of DiI-labelled aortic baroreceptor neurons with a holding potential at −60 mV. (**a**,**b**) Effect of T5E3 or preimmune IgG on the stretch-activated channel in neurons from wild-type mice (**a**) and *Trpc5*^*−/−*^ mice (**b**). (**c**) Summarized single-channel open probabilities (NP_o_) under different conditions. (**d**) Single-channel *I*−*V* relationship of the stretch-activated channel. (**e-h**) Whole-cell patch clamp recordings of mouse aortic baroreceptor neurons under different hydrostatic pressures. (**e**) Representative time course traces showing the whole-cell current change at ±80 mV upon pressure stimulation. (**f**) Representative whole-cell *I–V* relationship before (a) and after (b) hydrostatic pressure. a and b correspond to the time point shown in **e**. Ramp ramps from −80 mV to +80 mV with 500-ms duration were repeated every 1 s. (**g**) Summary of data showing the effect of T5E3 on the pressure-activated whole-cell current density at ±100 mV. Subtraction of currents under 0 mm Hg from those under 30 mm Hg yielded the pressure-activated currents (Δ current density). (**h**) Pressure-activated current density change in wild-type and *Trpc5*^*−/−*^ mice at ±80 mV. Abbreviations: *PreIgG*, preimmune IgG; *WT*, wild type; *KO*, *Trpc5*^*−/−*^. Mean±s.e.m. *P<0.05 as compared with no stretch in **c**, to preimmune IgG in **g** and to WT in **h**. ^#^*P*<0.05 as compared with WT before T5E3 at −30 mm Hg in **c**. ^&^*P*<0.05 as compared with WT before preIgG at −30 mm Hg in **c**. Statistical analysis was performed by Student’s *t*-test.

We further confirmed the baroreceptor function of TRPC5 with a knockout mouse model. T5E3-positive immunoreactive signals of TRPC5 were observed in nodose ganglion neurons of wild-type 129S1/SvImJ mice but not those of *Trpc5*^*−/−*^ mice (Fig. 5). The TRPC5 immunoreactivity located close to that of the neuronal marker PGP9.5 (Fig. 5). We used patch clamp to examine and compare the mechanosensitive channel in baroreceptor neurons derived from wild-type and *Trpc5*^*−/−*^ mice. Again, 20 μmol l^−1^ La^3+^ was included in the pipette solution to potentiate TRPC5 activity and to block other cation channels^20^. Stretch-activated channel activity was recorded at the single-channel level in the freshly dispersed mouse aortic baroreceptor neurons derived from wild-type 129S1/SvImJ mice but not in those derived from *Trpc5*^*−/−*^ mice (Fig. 6a–c). In time control experiments, we did not observe any time-dependent increase in channel activity in the absence of pressure. The stretch activation was reversible, disappeared upon the release of pressure (Supplementary Fig 5). The single-channel conductance was estimated to be ∼25±4 pS (Fig. 6d). The activity of the channel was inhibited by T5E3 (Fig. 6a,c). As an alternative approach, a high-speed pressure clamp was used to apply positive hydrostatic pressure to the inside of the neurons. Positive pressure instantly activated a whole-cell current in aortic baroreceptor neurons from wild-type mice but had little effect on those from *Trpc5*^*−/−*^ mice (Fig. 6e–h). Likewise, the pressure activation was reversible, disappeared upon the release of pressure (Supplementary Fig 5). The pressure-activated whole-cell current could be inhibited by T5E3 (Fig. 6g). *I–V* relationship of the pressure-activated whole-cell current was similar to that of TRPC5 (Fig. 6f)^20^.

**Figure 7:**
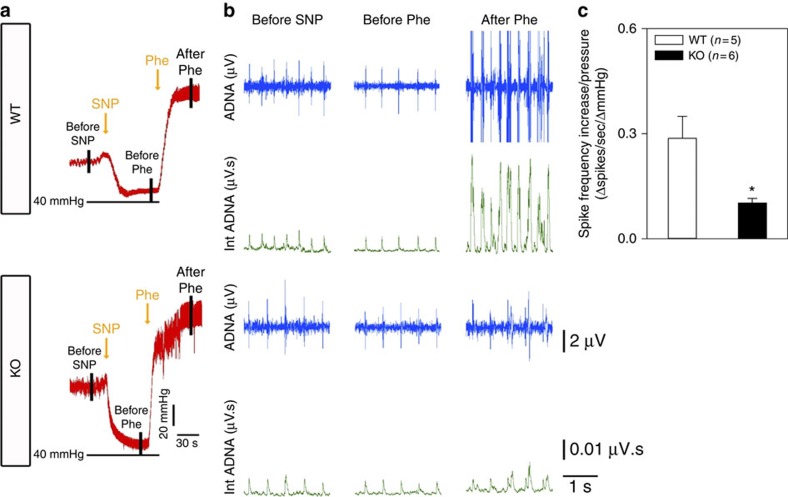
TRPC5 participates in the pressure-sensing of aortic baroreceptors. (**a**) Representative trace showing the procedures to alter blood pressure in 129S1/SvImJ mice. Sodium nitroprusside (SNP; 1.3 μg g^−1^ body weight) was intravenously injected, followed by phenylephrine infusion into femoral vein (*Phe*; 20 μg g^−1^ body weight), which raised the blood pressure. Action potentials at the left aortic depressor nerve were recorded continuously. (**b**) Representative traces of the pressure-induced changes in the spike frequency, aortic depressor nerve activity (ADNA) and Int ADNA in wild-type (*WT*) and *Trpc5*^*−/−*^ (*KO*) mice. (**c**) Data summary comparing the baroreceptor sensitivity (Δspikes s^−1^ ΔmmHg^−1^), which is the change in spike frequency per mmHg pressure increase. Mean±s.e.m. *P<0.05 as compared with WT, statistical analysis was performed by Student’s *t*-test.

The pressure-induced increase in aortic depressor nerve activity was compared between age-matched wild-type and *Trpc5*^*−/−*^ mice under anaesthetized conditions. Mice were intravenously injected with sodium nitroprusside (1.3 μg g^−1^ body weight), which reduced the blood pressure. Subsequent application of phenylephrine (20 μg g^−1^ body weight) elevated the blood pressure (Fig. 7a), which was higher in *Trpc5*^*−/−*^ mice than in wild-type mice (129±6 mm Hg (*n*=6) versus 106±10 mm Hg (*n*=5), *P*<0.05, by Student’s *t*-test). Elevation of arterial blood pressure caused a substantial increase in spike frequency and magnitude of Int ADNA in wild-type mice (Fig. 7b). The responses were much less pronounced in *Trpc5*^*−/−*^ mice (Fig. 7b). Aortic baroreceptor sensitivity (Δspikes s^−1^ ΔmmHg^−1^) was also clearly reduced in *Trpc5*^*−/−*^ mice when compared with the wild-type mice (Fig. 7c). Similarly, phenylephrine application also caused an increase in spike frequency and magnitude of integrated carotid sinus nerve activity in wild-type mice (Supplementary Fig. 6), and the responses were much less pronounced in *Trpc5*^*−/−*^ mice (Supplementary Fig. 6).

**Figure 8:**
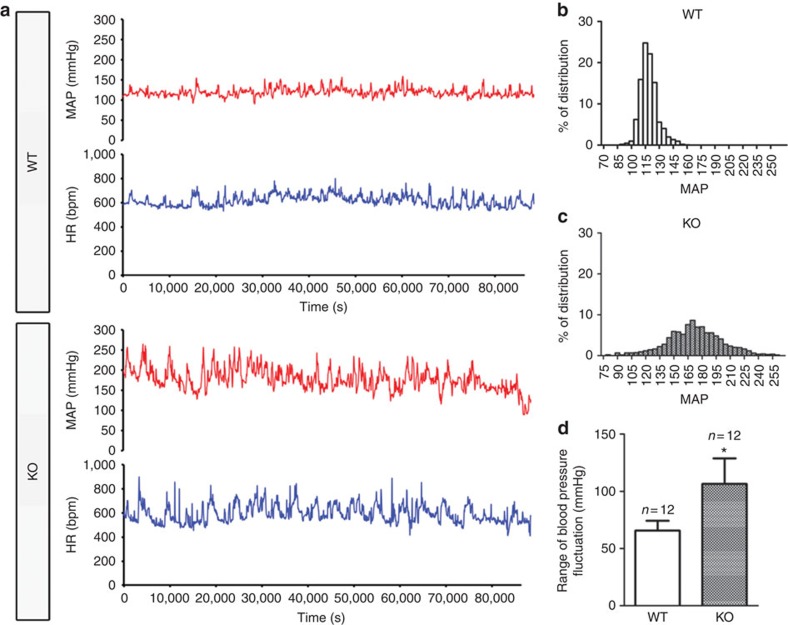
Freely moving conscious *Trpc5*^*−/−*^ mice show unstable mean arterial pressure. (**a**) Representative traces for 24 h recording of mean blood pressure and heart rate in 1 min time interval in a wild-type mouse (upper) and a *Trpc5*^*−/−*^ (lower) mouse. (**b**,**c**) Frequency distribution histogram of the mean arterial pressure in a 24-h period with the bandwidth of 15 mm Hg pressure range in wild-type and *Trpc5*^*−/−*^ mice. Shown was one representative pair from 12 pairs of mice. (**d**) Summarized data comparing the range of blood pressure fluctuation in 24 h between 12 pairs of wild-type and *Trpc5*^*−/−*^ mice. Mean±s.e.m. *n*=12. *P<0.05 as compared with WT, statistical analysis was performed by Student’s *t*-test.

Impaired baroreceptor function leads to dysregulation of blood pressure^3^. Therefore, we further examined the role of TRPC5 in daily blood pressure stability in freely moving, conscious mice by a telemetric device. The resting blood pressure was higher for *Trpc5*^*−/−*^ mice than for wild-type mice (158±26 mm Hg (*n*=12) versus 113±7 mm Hg (*n*=12), *P*<0.05, by Student’s *t*-test). Twenty-four hour continuous recordings of blood pressure and heart rate were made. Compared with the wild-type mice, *Trpc5*^*−/−*^ mice showed larger variations in mean arterial pressure (Fig. 8a–c) during the 24-h recording period. Mean arterial pressure values of *Trpc5*^*−/−*^ mice were distributed in a broader range than those of wild-type mice (Fig. 8b,c). The ranges of mean arterial pressure fluctuation in 24 h periods were 66 mm Hg (*n*=12) for normal mice and 106 mm Hg (*n*=12) for *Trpc5*^*−/−*^ mice, which represents a 60% increase in *Trpc5*^*−/−*^ mice (Fig. 8d). No difference was observed in central mediation of baroreflex (Supplementary Fig. 7a,b) and locomotion activity (Supplementary Fig. 7c,d) between wild-type and *Trpc5*^*−/−*^ mice.

**Figure 9:**
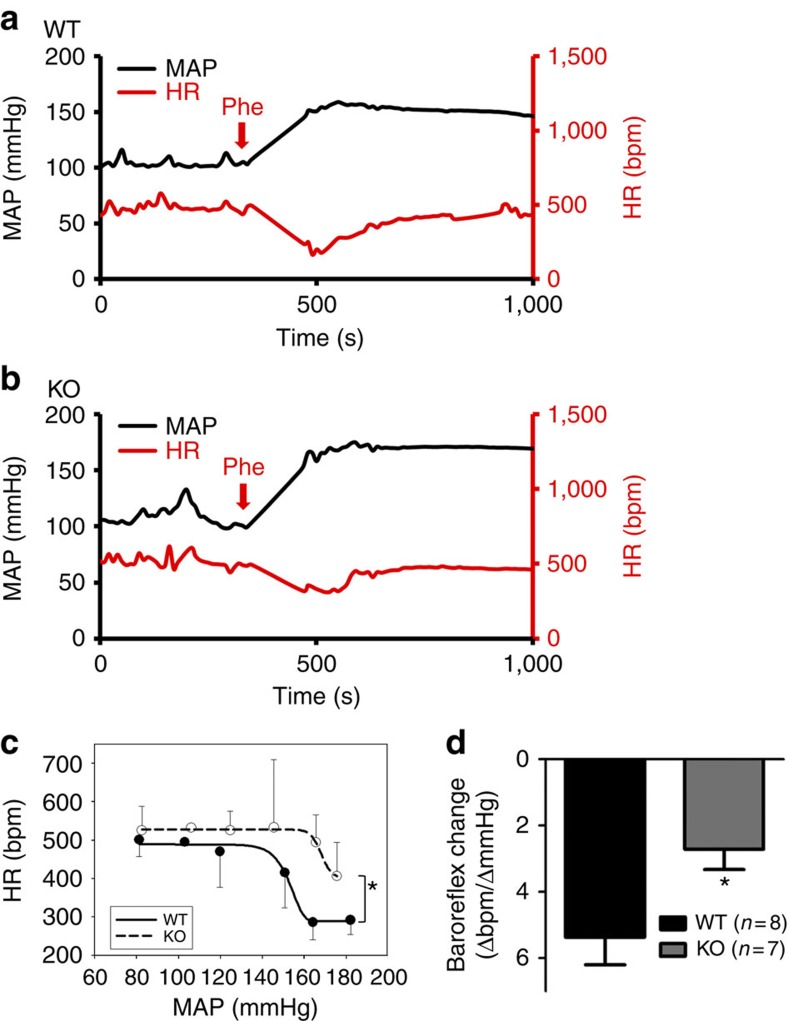
Conscious *Trpc5*^*−/−*^ mice show deficiency in baroreflex regulation of heart rate. (**a,b**) Representative traces showing the heart rate reduction (HR) in response to elevation of mean arterial pressure (MAP) induced by i.p. phenylephrine injection (*Phe*; 20 μg g^−1^ body weight) in wild-type (*WT;* **a**) and *Trpc5*^*−/−*^ (*KO*) mice (**b**). (**c**,**d**) Summarized data showing the baroreflex-mediated heart rate reduction in response to pressure increase. (**c**) The baroreflex curve over a range of MAP, statistical analysis was performed by two-way analysis of variance followed by Bonferroni post-test. (**d**) The overall baroreceptor sensitivity, which is the ratio of overall heart rate response divided by pressure change. Mean±s.e.m. *n*=7–8. *P<0.05 as compared with WT, statistical analysis was performed by Student’s *t*-test.

Baroreflex control of heart rate was also examined in conscious mice using the telemetric device. Bolus intraperitoneal injection of phenylephrine (20 μg g^−1^ body weight) caused an increase in blood pressure, which was accompanied by heart rate reduction (Fig. 9a,b). This baroreflex-mediated reduction in heart rate was significantly smaller in *Trpc5*^*−/−*^ mice than in wild-type mice (Fig. 9c,d).

## Discussion

Baroreceptors detect arterial blood pressure and participate in the baroreceptor reflex control of blood pressure^1^. However, the molecular identity of the mechanosensors in the baroreceptors remains elusive. Recently, we reported that TRPC5 is directly activated by membrane stretch^15^. In the present study, we provided comprehensive evidence for the critical role of a TRPC5-containing channel in mechanosensation of aortic baroreceptor neurons. These include: (i) A stretch-activated channel was recorded in the somata and neurite terminals of rat aortic baroreceptor neurons, the activity of which was blocked by T5E3 and T5DN. A similar stretch-activated channel was recorded in aortic baroreceptor neurons of wild-type mice but not *Trpc5*^*−/−*^ mice. Single-channel conductance of this channel resembled that of TRPC5. (ii) Hypoosmolarity activated the whole-cell currents in rat aortic baroreceptor neurons, which were potentiated by Gd^3+^ but were inhibited by T5E3, T5DN and 2-APB. The currents displayed a double-rectifying current–voltage relationship, which is typical of TRPC5. Hydrostatic pressure activated a similar current in the aortic baroreceptor neurons of wild-type mice but not *Trpc5*^*−/−*^ mice. (iii) Immunoblots, RT–PCR and immunostaining demonstrated the expression of TRPC5 in these neurons. Taken together, these data provide compelling evidence that TRPC5 participates in mechanosensing in aortic baroreceptor neurons. Previous studies showed that TRPC5 may exist as homomeric channels or it may heteromerize with other TRPC subunits, such as TRPC1 and TRPC4, to form heteromeric channels^23^,^24^. However, we found that knockdown of other TRPC isoforms (TRPC1, -C3, -C4 and -C6) did not alter the pressure-activated whole-cell current in aortic baroreceptor neurons. Further studies are still needed to determine the molecular composition of the TRPC5-containing channels in aortic baroreceptor neurons.

The role of TRPC5 in baroreceptor function was further examined in animal models *in vivo*. Arterial blood pressure *in vivo* is sensed by the baroreceptor nerve terminals located in the blood vessel adventitia. In our experiments, immunoreactive signals of TRPC5 proteins were detected at the baroreceptor terminals that innervate aortic arch adventitia. TRPC5 is a nonselective cation channel, the activation of which leads to membrane depolarization^25^. Presumably, stretch-induced activation of TRPC5 should depolarize the membrane potentials, causing increased action potential firings in sensory nerves and eventually baroreflex regulation of heart rate. Therefore, we examined the pressure-induced spike frequency increase in aortic depressor nerves as well as the baroreflex-mediated heart rate reduction. Transduction of lenti-T5DN on the rat left nodose ganglion was found to impair the pressure-induced spike frequency increase in the aortic depressor nerve as well as the baroreflex-mediated heart rate reduction. The pressure-induced spike frequency increases in aortic depressor nerve and carotid sinus nerve were also attenuated in *Trpc5*^*−/−*^ mice when compared with wild-type mice. In addition, *Trpc5*^*−/−*^ mice displayed a reduced baroreflex response in heart rate.

One of the most important physiological functions of the arterial baroreceptors is to regulate the stability of blood pressure^3^,^26^. Reports have shown that denervation of baroreceptors causes the blood pressure to become unstable^3^,^26^. Daily activities, such as postural changes from the supine to the upright position, eating, defecation and so forth, result in an elevated blood pressure in denervated dogs^3^,^26^. As a result, the fluctuation of arterial blood pressure becomes much greater in denervated animals^26^. In the present study, we found that the blood pressure of *Trpc5*^*−/−*^ mice varied considerably more than that of wild-type mice in a 24-h recording period. The ranges of mean arterial pressure fluctuation in the 24-h period were 1.6-fold broader in *Trpc5*^*−/−*^ mice than in wild-type mice. Taken together, these data support a key functional role of TRPC5 in baroreceptor mechanosensing and blood pressure stabilization *in vivo*.

However, the idea of TRPC5 involvement in baroreceptor mechanosensation does not exclude the participation of other mechanosensors in baroreceptor function. Mechanosensitive neurons in nodose ganglion are diverse; some give rise to myelinated baroreceptor afferent fibres while others give rise to un-myelinated baroreceptor fibres^17^,^27^; some are uni-modally mechanosensitive while others are bi-modally mechanosensitive and chemosensitive^27^; some demonstrate a pronounced depolarization while others only show a modest depolarization in response to mechanical stimuli^27^. Therefore, it is conceivable that multiple mechanosensors may exist in baroreceptor neurons, performing distinct functions in mechanosensing. Indeed, considerable baroreflex response of heart rate could still be observed in *Trpc5*^*−/−*^ mice (Fig. 9), suggesting the existence of some TRPC5-independent component(s) in baroreceptor mechanosensation. Several previous reports suggested possible contribution of ASIC2 (ref. 4), epithelial sodium channel γ-subunit^5^ and TRPV1 (ref. 6) in baroreceptor mechanosensing. It is possible that TRPC5 may function together with some of these components, contributing to mechano-stimulated membrane depolarization and consequential action potential firings. In addition, because of the scope of present study, we only explored the baroreceptor neurons without looking into the possibility of other mural cells acting as arterial baroreceptors. Further studies are needed to clarify these issues.

Cunningham *et al.* showed that Gd^3+^ inhibited the osmolarity-activated whole-cell current in aortic baroreceptor neurons^18^, which appears to contradict with our results (Fig. 2). We speculate that the discrepancy could be due to the differences in pipette/bath solutions and/or experimental procedures. We adopted a patch recording condition specifically suited for TRPC5 recording^13^,^15^,^20^. Cs-aspartate was used in the pipette and bath solutions to remove/reduce K^+^ and Cl^−^ currents. Gd^3+^ was included in the pipette before hypotonicity challenge. On the other hand, Cunningham *et al.* used physiological pipette and bath solutions^18^. It is well documented that Gd^3+^ could inhibit many ion channels including K^+^ channels, Ca^2+^ channels and anion channels; some of which are mechano-sensitive and others are mechano-insensitive^28^. Therefore, it is likely that Cunningham’s results may reflect Gd^3+^ inhibition on those channels. Along this line, it is conceivable that TRPC5 and a Gd^3+^-inhibitable channel could both play functional roles in baroreceptor mechanosensaton. Notably, ASIC2 and TRPV1 are Gd^3+^-inhibitable channels^29^,^30^.

In summary, we provide conclusive evidence that TRPC5 is a stretch-activated channel participating in baroreceptor mechanosensing and baroreflex regulation of blood pressure.

## Methods

### Animals

All animal experiments were conducted in accordance with the Guide for the Care and Use of Laboratory Animals issued by the US National Institute of Health (NIH) and approved by the Animal Experimentation Ethics Committee, Chinese University of Hong Kong. In all the animal studies, a minimum of five age-matched male adult pairs of 129S1/SvImJ mice (3–6 months) and Sprague–Dawley rats (180–220 g) were used.

### Total RNA isolation and RT–PCR

The total RNA was extracted from rat or mice left nodose ganglion using Trizol Reagent. First-strand cDNA was prepared using SuperScript II Reverse Transcriptase and oligo-dT primer. The PCR primers were as follows: TRPC5 forward primer 5′-AAGTTTCGAATTTGAGGAGCAGATG-3′, the reverse primer 5′-AATCTCTGATGGCATCGCACA-3′; and GAPDH forward primer 5′-CGAGAATGGGAAGCTTGTCATC-3′, the reverse primer 5′-CGGCCTCACCCCATTTG-3′. PCR reactions were performed with 2 μl first-strand cDNA, 5 μl of 10 × buffer, 1.5 μl of 50 mmol l^−1^ MgCl_2_, 0.2 mmol l^−1^ of dNTP, 1.0 μmol l^−1^ of primers and 2.5 U Taq DNA polymerase. Thirty-five cycles (94 °C for 1 min, 58 °C for 1 min, 72 °C for 1 min) were performed with a PCR machine (PTC-200, MJ Research).

### Immunoblots

Freshly isolated rat and mouse nodose ganglions were homogenized. The lysates of the ganglions or cultured cells were extracted with protein extraction buffer, which contains 150 mmol l^−1^ of NaCl, 20 mmol l^−1^ of Tris-Cl, 1 mmol l^−1^ of EDTA, 0.5% Triton X-100 and protease inhibitor cocktail (pH 7.4). Immunoblots were performed as described elsewhere^31^. Briefly, the lysate was centrifuged at 12,000 r.p.m. for 30 min at 4 °C. The supernatant was collected and the proteins were separated on a 7% SDS–PAGE gel and transferred to a polyvinylidene difluoride membrane. Proteins on the membrane were probed by the primary anti-TRPC5 antibodies (Alomone labs, 1:200) and/or anti-β-tubulin antibodies (Santa Cruz, 1:300) at 4 °C for 16 h. Immunodetection was accomplished with horseradish peroxidase-conjugated secondary antibodies (1:2,000), followed by reaction with ECL western-blot detection system. Full (uncropped) scans of western blots are available in Supplementary Fig. 8.

### Neuron culture and transfection

Nodose ganglion neurons were isolated from Sprague–Dawley rats and 129S1/SvImJ mice as described elsewhere with slight modifications^18^,^32^,^33^. Briefly, left nodose ganglion was dissected and digested for 1 h at 37 °C with DNAse I (0.1 mg ml^−1^), trypsin (1 mg ml^−1^) and collagenase IA (1 mg ml^−1^). The single neurons were dispersed by gentle trituration, followed by centrifugation. Mouse neurons were resuspended, plated in DMEM/F-12 media and then used within 24 h. Rat neurons were cultured in DMEM/F-12 media supplemented with 5% FBS and 100 ng ml^−1^ of 7S nerve growth factor (NGF). Cytosine arabinofuranoside (Ara-C; 10 μmol l^−1^) was included in culture medium for 4 days to inhibit the growth of dividing cells. The patch clamp experiments were performed on day 5. When needed, rat neurons were transiently transfected with T5DN or empty vector using electroporation, and were used for experiments 48–72 h afterward. T5DN was subcloned into lentiviral vector pRRL-cPPT-CMV-X-PRE-sin^34^. T5DN and *Trpc5*^*−/−*^ 129S1/SvImJ mice were gifts from D.E. Clapham (Harvard University)^23^,^35^.

For siRNA knockdown of TRPCs, the primary cultured rat nodose neurons were treated with scrambled siRNA or *Trpc*-targeting siRNA (ON-TARGETplus Rat siRNA—SMARTpool, Dharmacon) in individual wells on the first day after plating. Dharmacon DharmaFECT 1 reagent was used for transfection with 25 nmol l^−1^ siRNA per well. The cells were lysed and immunoblotting was performed 48–72 h after the siRNA treatment.

### Immunohistochemistry

The methods were similar to those described elsewhere^17^. Briefly, the left nodose ganglions, aortic depressor nerve and carotid sinus nerve were isolated from adult Sprague–Dawley rats and/or 129S1/SvImJ mice. The tissues were fixed with 3.7% formaldehyde, followed by embedment in Tissue-Tek O.C.T. medium (Sakura) and frozen by liquid nitrogen. The 10-μm cryosections were cut and incubated with or without the primary antibody mixture containing anti-TRPC5 rabbit-antibodies (Alomone Labs anti-TRPC5 for rats, 1:100; or T5E3 for mice, 1:100), anti-PGP9.5 goat-antibodies (Abcam, 1:100), and/or anti-MBP (myelin basic protein) mouse-antibody (Millipore, 1:100) at 4 °C overnight. After washing with PBS, the sections were incubated for 1 h at room temperature with secondary donkey anti-rabbit/goat/mouse IgG (1:500) conjugated to Alexa Fluor 488/546. For cultured nodose neurons, the fixed cells were probed with T5E3 (1:100, extracellular epitope). The immunofluorescence signals were recorded by Fluoview FV1000 confocal microscopy system (Olympus). T5E3 antibody was raised in rabbits using the strategy developed by Xu *et al.*^21^

The rat aortic arch adventitia, which contains the baroreceptor nerve terminals, was dissected out and fixed in 3.7% formaldehyde overnight as described elsewhere^17^. The adventitia was permeabilized by 1% Triton X-100 at 4 °C for 3 h. The procedures for immunostaining were the same as described in the paragraph above. Series of Z-images were captured and stacked.

### DiI-labelling of aortic baroreceptor neurons

The surgical procedures were similar to those described elsewhere^18^,^32^. Briefly, rats and mice were anaesthetized with pentobarbital sodium and ketamine/xylazine cocktail, respectively. Nerves in the left cervical area were exposed. 3–4 mm of the aortic depressor nerve was carefully detached from surrounding tissues. DiI crystals were applied around the left aortic depressor nerve with a parafilm underneath, and the area was wrapped by a rapid-curing silicone gel (Kwik-Sil, World Precision Instruments). The incision was sutured afterward. The animals were recovered for 5–7 days to allow DiI dye to diffuse retrogradely along the aortic depressor nerve to the soma located in nodose ganglion.

### Patch clamp

Whole-cell voltage clamp and single-channel recordings were achieved by an EPC9 patch clamp amplifier (HEKA). In whole-cell recording, the holding potential was 0 mV, and *I*–*V* relationship was obtained using a ramp protocol from −100 mV to +100 mV with 500 ms duration and repeated every 1 s. The maximal increase in electrical currents in response to hypoosmolarity was plotted in Fig. 2. Hydrostatic pressure was applied to the inside of cells using a high-speed pressure clamp (HSPC-1, ALA Scientific Instruments). The whole-cell currents were normalized by cell capacitance into current density (pA/pF). QX314 (5 mmol l^−1^) was included to inhibit voltage-gated Na^+^ channels and prevent action potential firing. When needed, the cells were pretreated with T5E3 (15 μg ml^−1^) or preimmune IgG (15 μg ml^−1^) for 45 min, or with 2-APB (75 μmol l^−1^) or Gd^3+^ (20 μmol l^−1^) for 15 min before the recording. In single-channel recordings, a U-shaped water-filled hydraulic pressure system was used to generate negative pressure. The single-channel activity was recorded for 40–60 s after stretch and NP_o_ was calculated from a 30-s period with high activity. For single-channel experiments with T5E3 or preimmune IgG, the patch pipettes were backfilled with T5E3 (15 μg ml^−1^) or preimmune IgG (15 μg ml^−1^) using a two-step protocol as shown in the schematic diagram in Fig. 1d^22^. Briefly, the lower half of the glass pipette was filled with a pipette solution without T5E3 or PreIgG, whereas the upper half was filled with the pipette solution containing T5E3 or PreIgG. In this design, immediately after seal formation, there were no contact between antibodies and membrane patch. After a few seconds, the antibodies in the upper part would gradually diffuse to the pipette tips, making contact with the membrane patches. The negative pressure was applied multiple times, each lasted ∼30 s, during which the single channel activity was recorded and NP_o_ was calculated. The total recording period lasted for ∼15 min. T5E3 inhibition was observed 5–10 min after the patch seal formation. All currents were sampled at 50 kHz and filtered at 5 kHz. The junction potentials were corrected. The whole-cell and single-channel current data were analysed with PulseFit and TACFit software, respectively. All experiments were performed at room temperature.

For whole-cell recording, the pipette solution contained in mmol l^−1^: 130 Cs-aspartate, 1 MgCl_2_, 5 Na_2_ATP, 5.9 Ca-gluconate, 10 EGTA, 10 HEPES, pH 7.2, with CsOH. Free Ca^2+^ was ∼200 nmol l^−1^. The bath solution was either the isotonic solution or the hypotonic solution or a Cs-containing physiological saline. Isotonic solution contained in mmol l^−1^: 65 Na-aspartate, 5 KCl, 1 CaCl_2_, 1 MgCl_2_, 10 HEPES, 10 glucose, 140 mannitol, pH 7.4, with NaOH, ∼300 mOsm. Hypotonic solution contained in mmol l^−1^: 65 Na-aspartate, 5 KCl, 1 CaCl_2_, 1 MgCl_2_, 10 HEPES, 10 glucose, pH 7.4, with NaOH. The Cs-containing physiological saline contained in mmol l^−1^: 130 Na-aspartate, 5 Cs-aspartate, 5 KCl, 1 CaCl_2_, 1 MgCl_2_, 10 glucose, 10 HEPES, pH 7.4, with NaOH. 20 μmol l^−1^ of LaCl_3_ was included in the bath solution. For inside-out single-channel recording, the pipette solution was normal physiological saline solution, which contained in mmol l^−1^: 140 NaCl, 5 KCl, 1 MgCl_2_, 1 CaCl_2_, 10 glucose, 10 HEPES, pH 7.4. The bath solution was in mmol l^−1^: 140 Cs-aspartate, 5 CsCl, 1 MgCl_2_, 1 CaCl_2_, 10 HEPES, pH 7.4, with CsOH. In some experiments, the bath solution was a high K^+^ solution, which contained in mmol l^−1^: 130 K-aspartate, 5 NaCl, 1 MgCl_2_, 1 CaCl_2_, 10 glucose, 10 HEPES, pH 7.4, with KOH. 20 μmol l^−1^ LaCl_3_ was included in the pipette solution except for the Gd^3+^ series of experiments. For inside-out single-channel recordings, free Ca^2+^ in bath solution was adjusted to ∼500 nmol l^−1^ for the optimal TRPC5 activity^36^.

### Lentiviral transduction and detection of action potential

Left nodose ganglion of male Sprague–Dawley rats was exposed. Lentiviruses (∼1.5 × 10^7^ copies of lenti-T5DN, lenti-GFP or lenti-vector) were topically applied to the left nodose ganglion. The recordings were carried out on day 6 after lentiviral transduction. Blood pressure was continuously measured by a pressure transducer cannulated to right common carotid artery. A segment of abdominal aorta superior to two renal arteries was gripped by an inflatable cuff, which served as a vascular occluder^37^. Arterial blood pressure was raised by gradually inflating the cuff with a pump^37^. In mouse experiments, the blood pressure was elevated by injecting phenylephrine (20 μg g^−1^ body weight) into femoral vein. To record spike frequency in aortic depressor nerve, the left aortic depressor nerve was isolated from its surrounding, and gently placed on a bipolar silver electrode, which was connected to an amplifier (Model 1700, A-M Systems Inc.)^38^. The nerve activity was amplified 10,000 times and filtered through a bandpass between 0.1 and 5 kHz. The signal was continuously recorded and the data were analysed by Clampfit 9.0 software (Axon Instruments). Integrated nerve activity was the absolute integral value of the recorded nerve activity with time constant of 0.01 s calculated by LabChart (AD Instruments).

### Telemetric recording of blood pressure

The method was modified from a previous protocol^39^. Anaesthesia of 129S1/SvImJ mice was induced and maintained with 3% and 1.5% isoflurane, in a 3:1 O_2_ to air ratio, respectively. The catheter was inserted into the right common carotid artery and advanced in order to place the blood pressure sensor in aorta for blood pressure measurement. The telemetric device, HD-X11, was embedded around the abdominal region subcutaneously. The animals were allowed to recover for at least a week before recording. Twenty-four hour telemetric recording of blood pressure and heart rate were performed in the conscious animals with DSI Dataquest A.R.T. telemetry system (Data Science International). The data were analysed using Spike2 (version 6.06, Cambridge Electronic Design). Average mean arterial blood pressure and heart rate in 1 min time interval were plotted continuously for 24 h from 10:00 hours to 10:00 hours of the next day. The mean arterial blood pressure was calculated based on the standard equation of MAP=(1/3 × SBP)+(2/3 × DBP). Frequency distribution histogram of the mean arterial blood pressure recorded in a 24-h period was plotted with the bandwidth of 15 mm Hg pressure range. The highest and lowest 2.5% of the recorded blood pressure values were discarded in order to remove possible noise. After that, the range of blood pressure variation was obtained by subtracting the lowest leftover value from the highest leftover value of the mean arterial pressure. Baroreflex control of heart rate in mice was investigated following i.p. injection of phenylephrine (20 μg g^−1^ body weight). Blood pressure and heart rate were recorded as above.

### Evaluation of the central mediated baroreflex response

Wild-type and *Trpc5*^*−/−*^ 129S1/SvImJ mice were anaesthetized with ketamine/xylazine cocktail as above. Aortic depressor nerve was stimulated by step increases in electrical stimulation (frequencies from 2.5 to 40 Hz) with pulse of 2 ms and 10 V for 20 s (ref. 4). The reduction in mean arterial pressure and heart rate was recorded.

### Measurement of locomotion activity

Open field test was performed to evaluate the locomotion activity of wild-type and *Trpc5*^*−/−*^ 129S1/SvImJ mice within a 24-h period. Mice were habituated to the environment for 10 min before the 24-h recording with chew and drink supplied. Total travel distance and immobile time were analysed.^40^

### Statistics

Student’s *t*-test was used for two group comparison. Two-way analysis of variance followed by Bonferroni post-test was used for comparison of multiple groups in baroreflex curve.

### Data availability

The authors declare that all other relevant data supporting the findings of this study are available on request.

## Additional information

**How to cite this article:** Lau, O.-C. *et al.* TRPC5 channels participate in pressure-sensing in aortic baroreceptors. *Nat. Commun.* 7:11947 doi: 10.1038/ncomms11947 (2016).

## Supplementary Material

Supplementary Information Supplementary Figures 1-8
