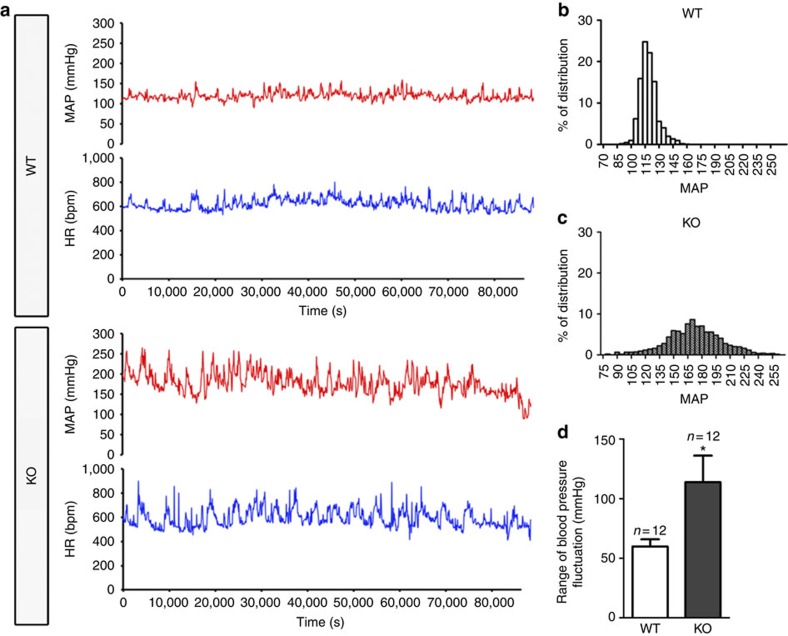# Author Correction: TRPC5 channels participate in pressure-sensing in aortic baroreceptors

**DOI:** 10.1038/ncomms16184

**Published:** 2018-03-23

**Authors:** On-Chai Lau, Bing Shen, Ching-On Wong, Yung-Wui Tjong, Chun-Yin Lo, Hui-Chuan Wang, Yu Huang, Wing-Ho Yung, Yang-Chao Chen, Man-Lung Fung, John Anthony Rudd, Xiaoqiang Yao

Nature Communications 7: Article number: 11947; DOI: 10.1038/ncomms11947 (2016); Published online: 07 14 2016; Updated: 03 23 2018

It has come to our attention that errors were made in measurements of resting blood pressure reported in this article, in Fig. 8 and in the ‘Results’ section entitled ‘Deficiency of baroreceptor function in Trpc5 knockout (*Trpc5*^*−/−*^) mice’. Two of the twelve measurements for *Trpc5*^*−/−*^ mice exceeded physiologically realistic values (277.3 and 390.5 mm Hg), and we assume were a result of technical errors in blood pressure recording. Upon removal of these two outliers and their corresponding control measurements, and replacement with resting blood pressure measurements from two additional pairs of mice, we no longer find a statistically significant difference in the resting mean arterial pressure between *Trpc5*^*−/−*^ and wild-type mice (121±8 mm Hg, *n*=12 for *Trpc5*^*−/−*^mice versus 105±4 mm Hg, *n*=12 for wild-type mice, *P*>0.05 by Student’s *t*-test). We can therefore no longer conclude that *Trpc5* knockout increases basal blood pressure. However, a statistically significant difference in range of blood pressure variation over 24 h remains (60±6 mm Hg, *n*=12 for wild-type mice and 114±22 mm Hg, *n*=12 for *Trpc5*^*−/−*^ mice, *P*<0.05 by Student’s *t*-test). These errors therefore do not affect our conclusion that *Trpc5* knockout results in blood pressure instability.

An updated version of Fig. 8d, which includes the above data, is presented below as Fig. 1. The error has not been corrected in the original version of the Article. Furthermore all resting mean arterial blood pressure data from the two mouse cohorts are presented as Supplementary Data 1 associated with this Author Correction.

We thank Pratish Thakore, Prof Susan Brian and Prof David Beech for their Correspondence, which highlights this issue^1^. In an associated Reply, we also respond in more detail to the criticisms raised^2^.

1. Thakore, P., Brain, S. D. & Beech, D. J. Challenging a proposed role for TRPC5 in aortic baroreceptor pressure-sensing. *Nat. Commun*. https://doi.org/10.1038/s41467-017-02703-w (2018).

2. Lau, O.-C. *et al*. Reply to ‘Challenging a proposed role for TRPC5 in aortic baroreceptor pressure-sensing’. *Nat. Commun*. https://doi.org/10.1038/s41467-017-02704-9 (2018).

## Supplementary Material

Supplementary Data 1 Three raw data sets of mean arterial blood pressure measurements. The left columns contain the measurements of original 12 pairs with two outliers labelled in red crossed out. The middle columns contain the measurements of original 10 pairs with two outliers removed. The right columns contain the measurement of final 12 pairs with two outliers removed and two new pairs added. WT, wild-type mice; KO, *Trpc5*^*−/−*^ mice.

## Figures and Tables

**Figure 1:**