# Supplementary material for: TRPC5 channels participate in pressure-sensing in aortic baroreceptors
Source: Nat Commun. 2016 Jul 14;7:11947. doi: 10.1038/ncomms11947 (PMC4947175; doi:10.1038/ncomms11947)
Supplement: Supplementary Information — Supplementary Figures 1-8 [file ncomms11947-s1.pdf]

Supplemental Figure 1

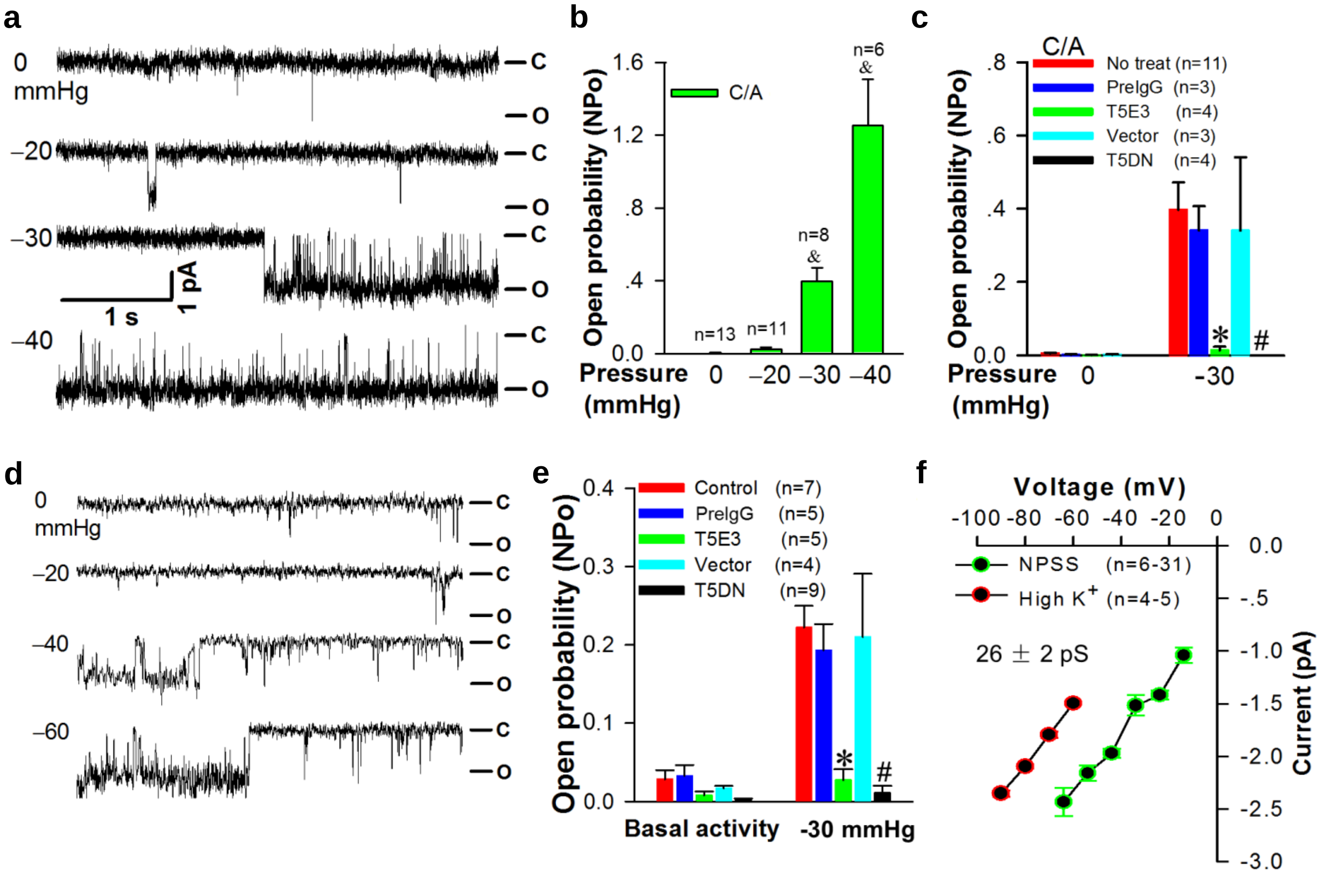

Supplementary Figure 1. A TRPC5-like channel in the neurites and somata of aortic baroreceptor neurons.

Cell-attached patch recordings from the neurite terminals **(a-c)** and the somata **(d-f)** of DiI-positive aortic baroreceptor neurons in primary culture. **(a,d),** Representative single-channel current traces under negative pressures (0, -20, -30 and -40 mmHg) with -60 mV holding potential. **(b,c,e),** Single-channel open probabilities (NPo) calculated from different patches, showing the channel activation by stretch **(b,c,e)**, inhibitions by T5E3 and T5DN **(c,e)**. **(f)** Single-channel *I-V* relationships of the stretch-activated channels in cell-attached mode. The bath solution was normal saline solution (NPSS, green circle) or 130 mmol L^{-1} K^+ extracellular solution (high K^+, red circle). Mean±s.e.m. $^{\&}P < 0.05$ as compared to 0 mmHg in **b**. $^{*}P < 0.05$ as compared to preimmune IgG (PreIgG) in **c** and **e**. $^{\#}P < 0.05$ as compared to vector in **c** and **e**. Statistical tests were performed by Student's *t*-test.

Supplemental Figure 2

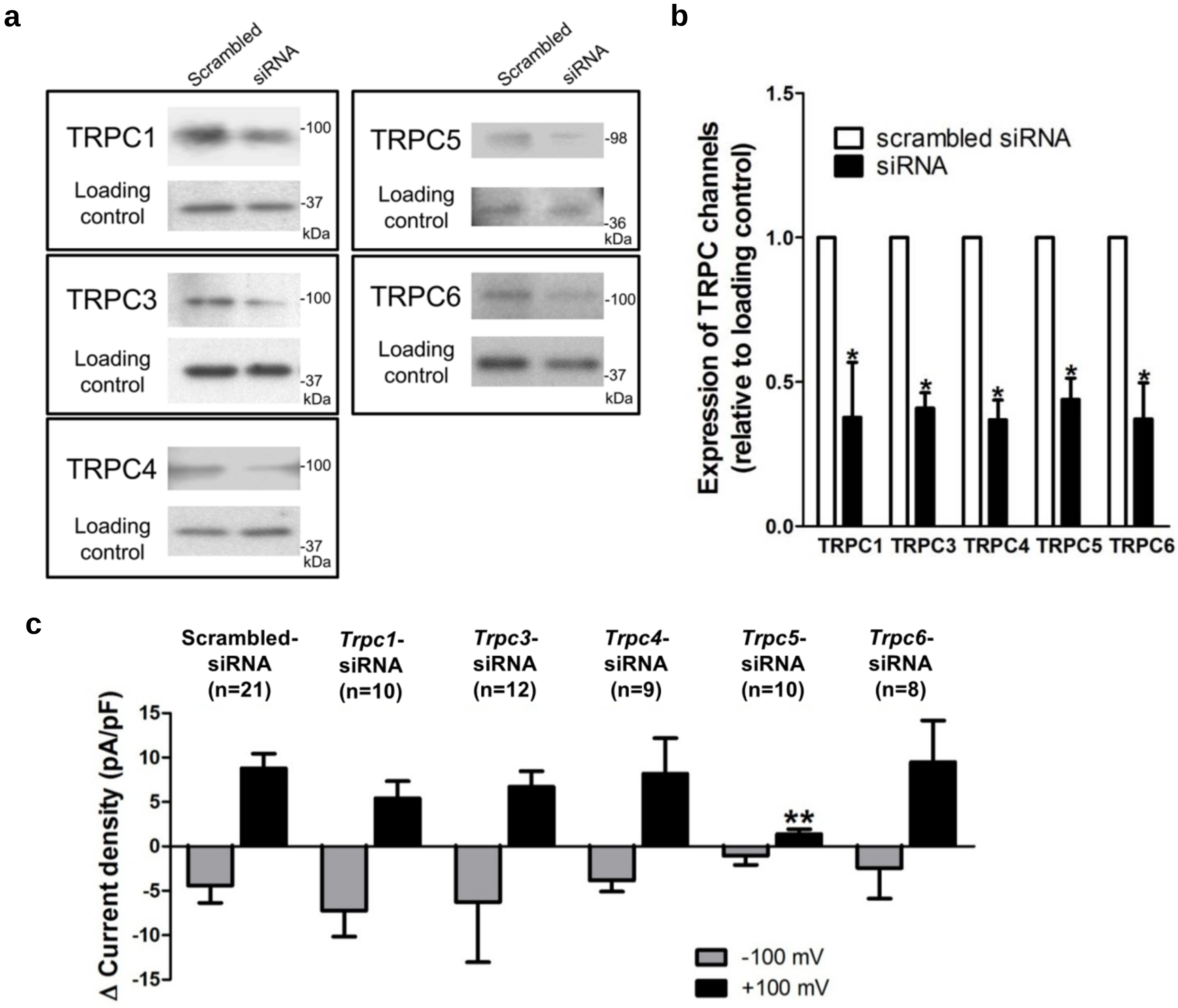

Supplementary Figure 2. Contribution of TRPC isoforms in the pressure-activated whole cell current in nodose neurons.

(**a-b**) Representative Western blot images (**a**) and data summary (**b**) showing the knockdown efficiency of individual *Trpc*-siRNAs. The primary cultured rat nodose neurons were treated with scrambled siRNA or individual *Trpc*-targeting siRNA (ON-TARGETplus Rat siRNA–SMARTpool, Dharmacon). β-actin was used as the loading control for TRPC3-6 and GAPDH was used as the loading control for TRPC1. Mean ± s.e.m (n = 3). *$P < 0.05$ as compared to scrambled siRNAs, by Student's *t*-test. (**c**) Effect of individual TRPC knockdown on the pressure-activated whole-cell current. The cells were stimulated by hydrostatic pressure of 30 mmHg. The pressure-activation currents at +100 and -100 mV were recorded. Mean ± s.e.m. **$P < 0.01$ as compared to scrambled siRNA, by Student's *t*-test.

Supplemental Figure 3

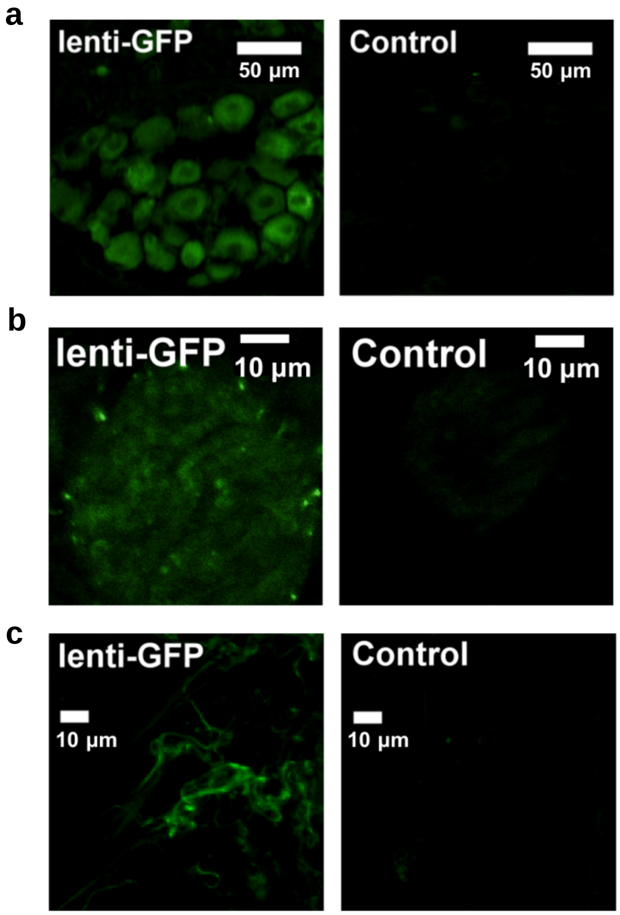

Supplementary Figure 3. Effectiveness of lentiviral vector-mediated transgene expression *in vivo*.

The rat left nodose ganglion neurons were transduced with lenti-GFP at day 0, followed by detection of GFP fluorescence at day 6 in the tissues. **(a-c)** Representative images (n = 3) showing the expression of GFP in the left nodose ganglion neurons **(a)**, aortic depressor nerve **(b)** and aortic arch baroreceptor terminals **(c)** from rats transduced with or without (labeled as control) lenti-GFP.

Supplemental Figure 4

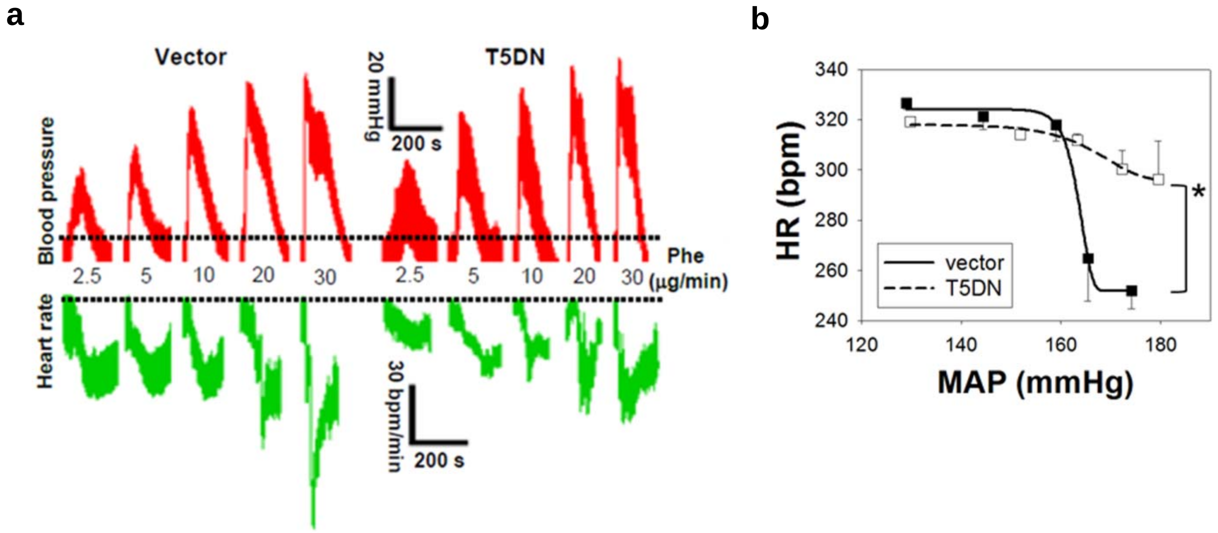

Supplementary Figure 4. Transduction of lenti-T5DN impaired the baroreflex-mediated heart rate response.

(a) Representative traces showing that intravenous infusion of phenylephrine (2.5 to 30 μg min^{-1}) resulted in an increase in arterial blood pressure (red traces) and a reflex reduction of heart rate (green traces). Left panel, rats transduced with lenti-vector; Right panel, rats transduced with lenti-T5DN. The dashed line indicates the baseline level of mean arterial pressure and heart rate. Heart rate is expressed in beats per min (bpm). **(b)** Summarized data showing the baroreflex-mediated heart rate reduction in response to the pressure increase. Mean ± s.e.m (n = 8). *P < 0.05 as compared to vector, by two-way ANOVA followed by Bonferroni post-test.

Supplemental Figure 5

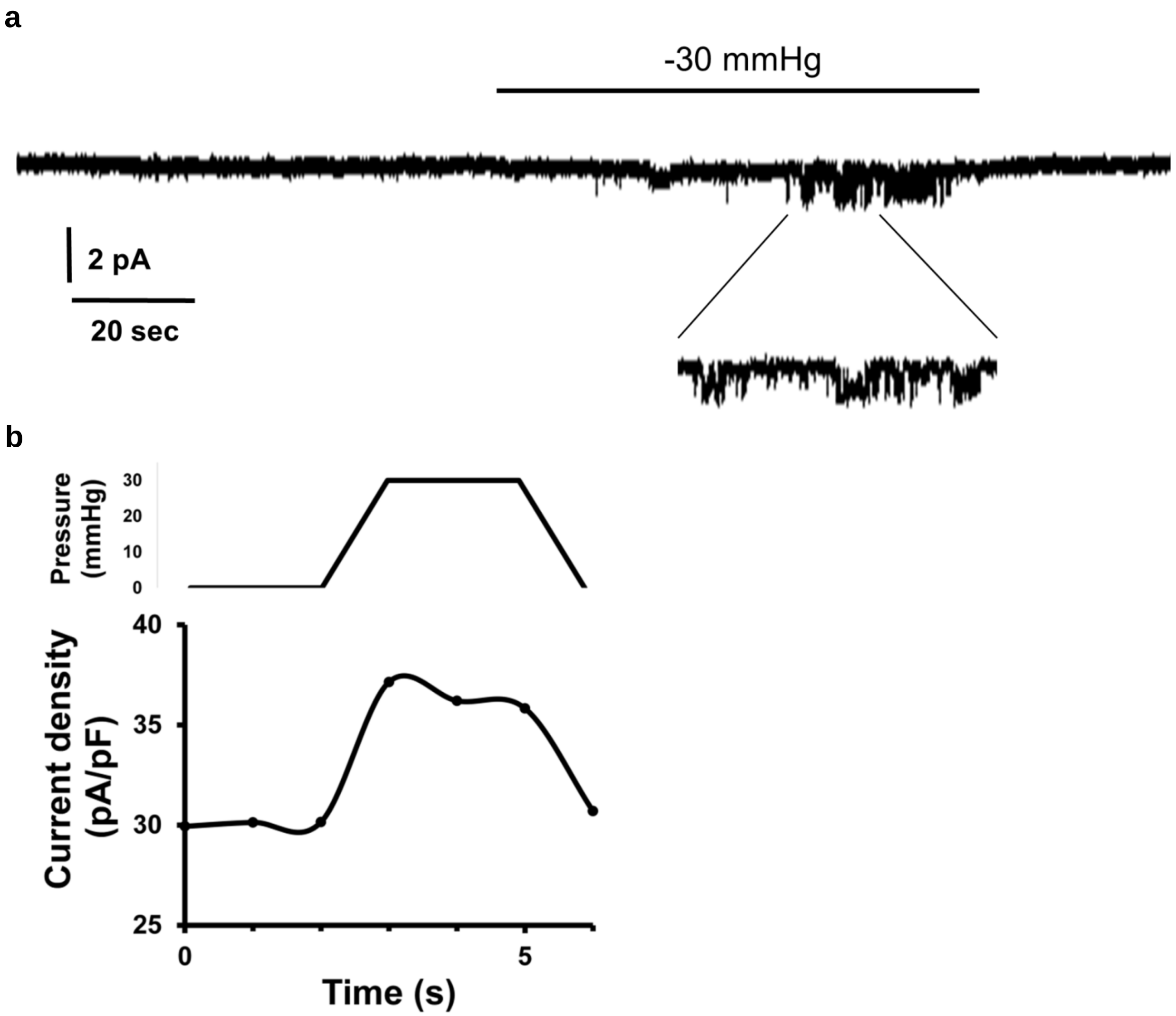

Supplementary Figure 5. Reversibility of the pressure-activated channel activity.

(**a**) Representative time course trace of pressure-activated single channel activity in mouse primary cultured baroreceptor neurons (n = 4). Hydrostatic pressure of -30 mmHg was applied as indicated by the horizontal bar. (**b**) Representative time course trace of pressure-activated whole-cell current in rat primary cultured baroreceptor neurons (n = 21). The upper panel shows the profile of hydrostatic-pressure exerted onto the cell, while the lower panel displays the corresponding whole-cell current at +100 mV at 1 sec interval.

Supplemental Figure 6

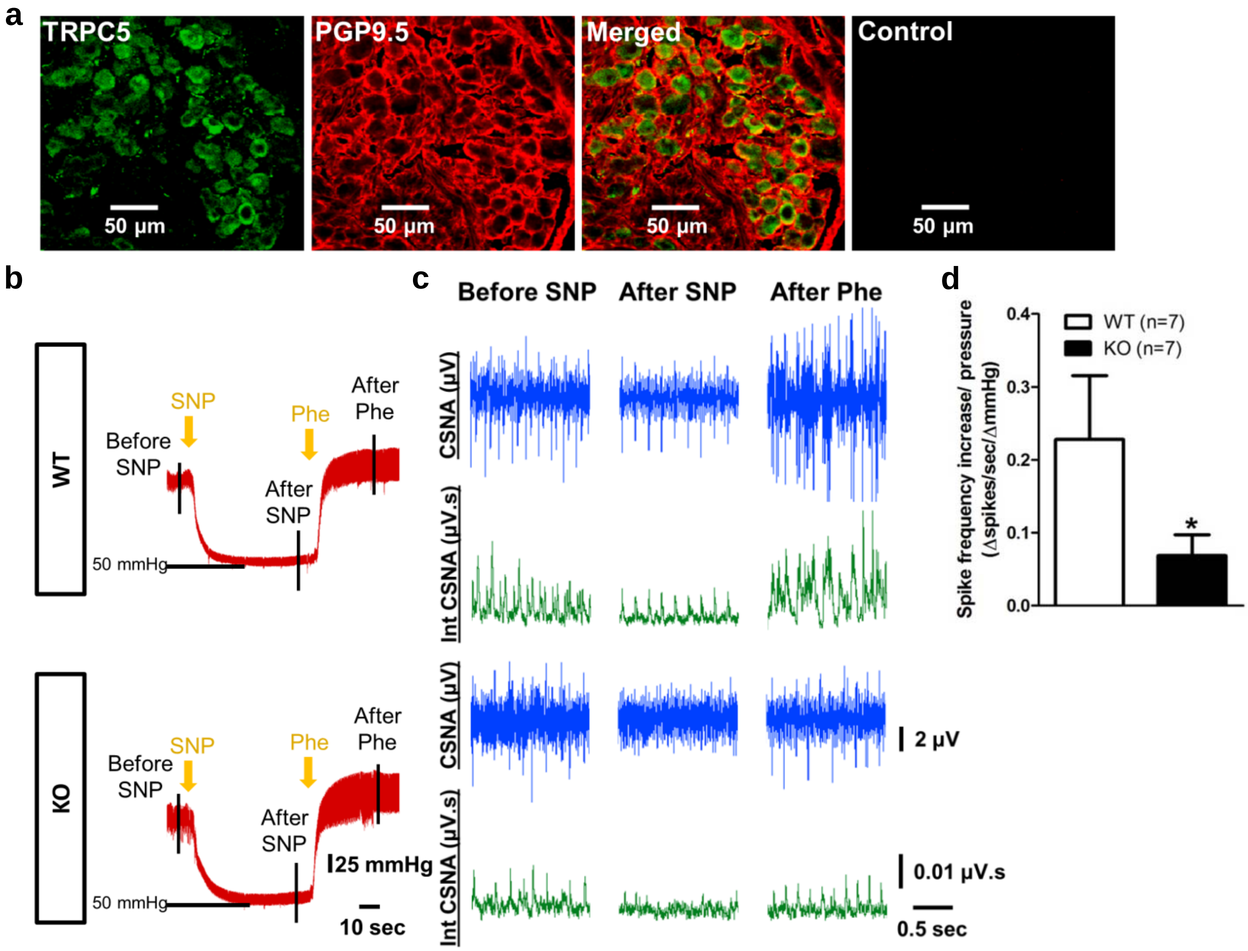

Supplementary Figure 6. TRPC5 participates in the pressure-sensing of carotid baroreceptor in wild-type mice.

(a), Representative images showing the immunoreactivity to TRPC5 (green), a neuronal marker PGP9.5 (red) at left petrosal ganglion (*n* = 3). Controls were without primary antibodies. Also shown were the merged images of TRPC5 and PGP9.5. (**b-d**) compared the pressure-induced action potential firings in carotid sinus nerve between wild-type (*WT*) and *Trpc5*$^{-/-}$ (*KO*) mice**.** **(b),** Representative trace showing the procedures to alter blood pressure in 129S1/SvImJ mice. Sodium nitroprusside **(***SNP*; 1.3 μg g^{-1} body weight**)** was intravenously injected, followed by phenylephrine infusion into femoral vein (*Phe*; 20 μg g^{-1} body weight), which raised the blood pressure. Action potentials at the left carotid sinus nerve were recorded continuously. **(c),** Representative traces of the pressure-induced changes in the spike frequency, carotid sinus nerve activity (CSNA) and Int CSNA in wild-type (*WT*) and *Trpc5*$^{-/-}$ (*KO*) mice. **(d),** Data summary comparing baroreceptor sensitivity (Δspikes sec^{-1} ΔmmHg^{-1}), which is the change in spike frequency per mmHg pressure increase. Mean ± s.e.m. *P < 0.05 as compared to WT, by Student's *t*-test.

Supplemental Figure 7

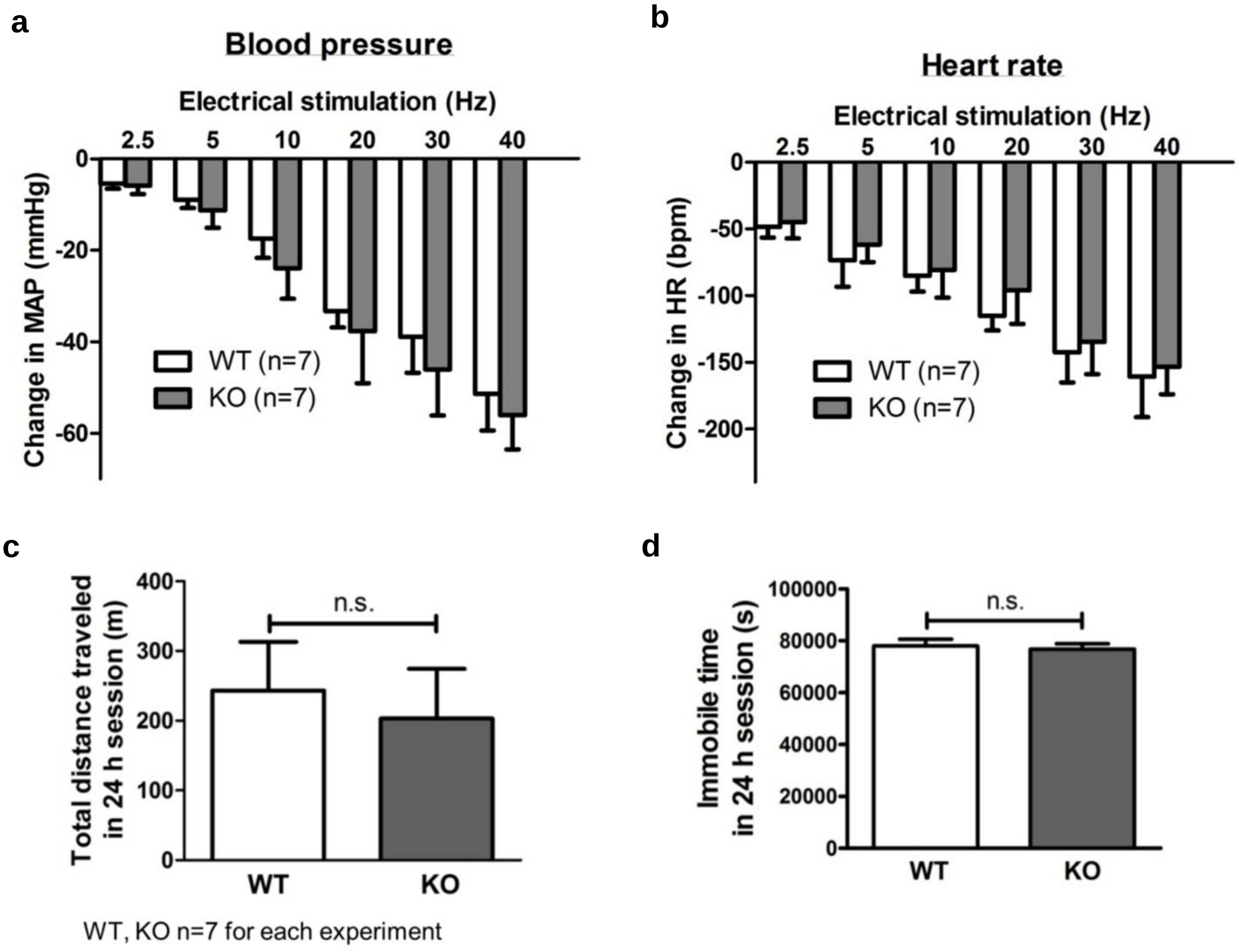

Supplementary Figure 7. *Trpc5*$^{-/-}$ mice display normal central mediation of baroreflex response and locomotion activity.

(**a and b)** Central-mediated baroreflex changes in mean arterial blood pressure (MAP in **c**) and heart rate (HR in **d**) in response to graded frequency (2.5 to 40 Hz) of electrical stimulation (10 V) on aortic depressor nerve in wild-type and *Trpc5*$^{-/-}$ mice. **(c and d)** Locomotion activity was assessed by open field test. Mice were habituated to the environment for 10 min prior to a 24 hr recording period with chew and drink supplied. Shown were total distance traveled (**a**) and immobile time (**b**) by wild-type and *Trpc5*$^{-/-}$ mice in the 24 hr session. Abbreviations: *WT*, wild-type; *KO*, *Trpc5*$^{-/-}$. Mean ± s.e.m (n = 7), $P > 0.05$, n.s., by Student's *t*-test.

Supplemental Figure 8

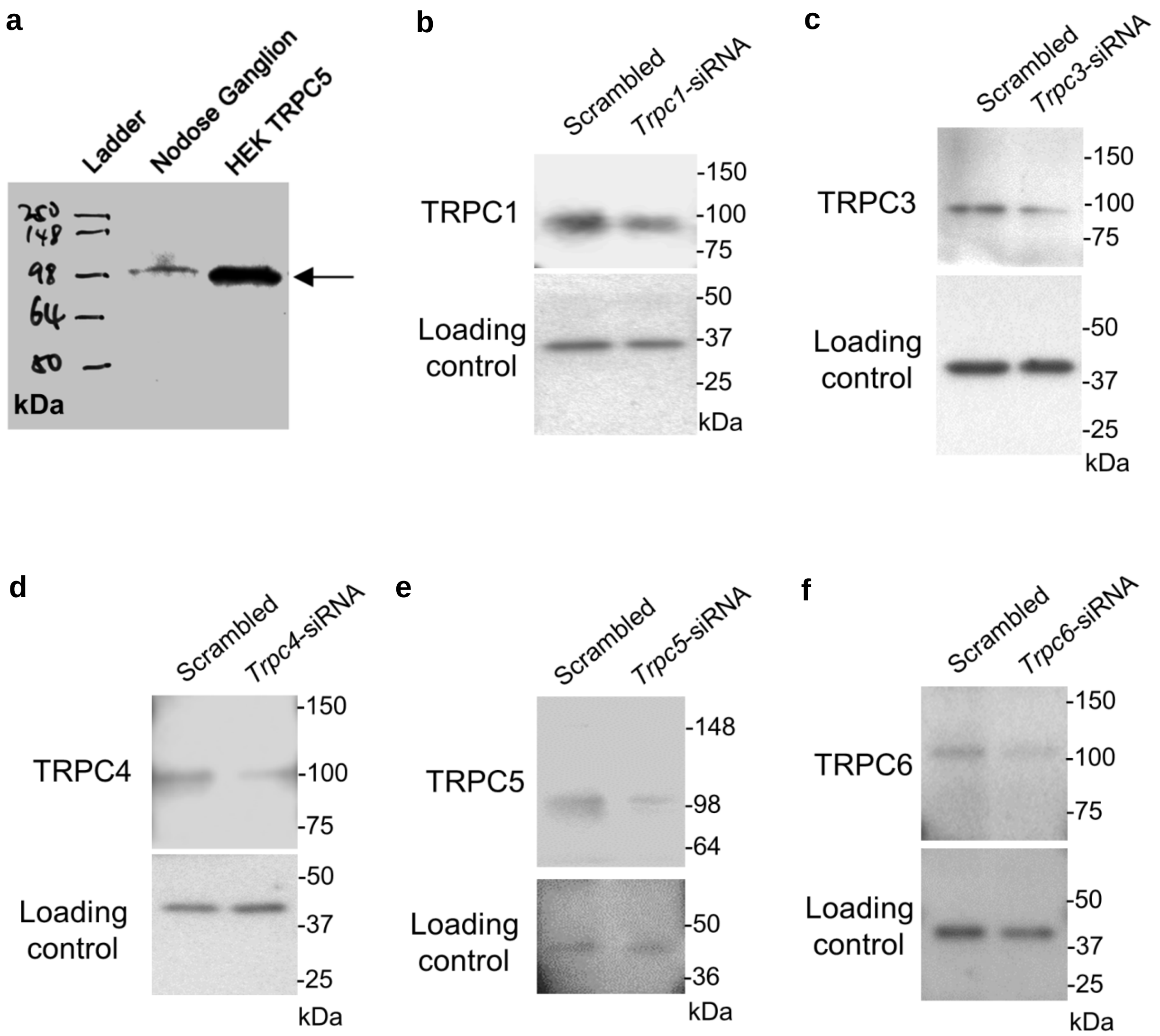

Supplementary Figure 8. Uncropped Western blot images.

(a) Uncropped Western blot images for Fig. 3b. **(b-f)** Uncropped Western blots images for Supplementary Fig. 2.
